# Ten quick tips for navigating intellectual property in FAIR educational resources

**DOI:** 10.1371/journal.pcbi.1013208

**Published:** 2025-07-08

**Authors:** Eva Maria Funk, Ulf Toelch, Rebecca Ludwig, Silke Kniffert

**Affiliations:** 1 Berlin Institute of Health (BIH) at Charité—Universitätsmedizin Berlin, QUEST Center for Responsible Research, Berlin, Germany; 2 EATRIS European infrastructure for translational medicine, Amsterdam, The Netherlands; SIB Swiss Institute of Bioinformatics, SWITZERLAND

## Abstract

Open Educational Resources (OER) offer a transformative approach to education by providing freely accessible learning materials. However, creating OER that meet the FAIR principles—findable, accessible, interoperable, and reusable—while navigating the complexities of copyright law presents unique challenges. This paper serves as a practical guide for educators and institutions wishing to develop and share FAIR-compliant OER while respecting intellectual property rights. It presents 10 quick tips that guide the process of developing legally secure FAIR OER from planning to creation to publication, including considerations for AI-generated materials. These quick tips cover critical aspects such as understanding licensing options, cataloguing resources to ensure comprehensive attribution, and effectively managing copyright concerns. By following these guidelines, educators can improve the discoverability and reusability of their materials, streamline the creation process, and contribute to a more open and collaborative educational landscape. This comprehensive approach not only supports the widespread dissemination of knowledge but also empowers educators to innovate and share resources effectively, fostering a global community of open science and education practice, while adhering legal regulations.

## Introduction

Education lays the foundation for the next generation of scientists, providing them with the knowledge, skills, and ethical framework necessary to contribute meaningfully to scientific progress and to address the challenges facing society. In recent years, the integration of digital resources has significantly shaped education, with Open Educational Resources (OER)—publicly available materials ranging from textbooks to multimedia that can be freely accessed, modified, and distributed—becoming increasingly important [1]. OER play a crucial role in complementing traditional teaching methods and fostering a more dynamic, collaborative, and accessible educational environment.

**Fig 1:**
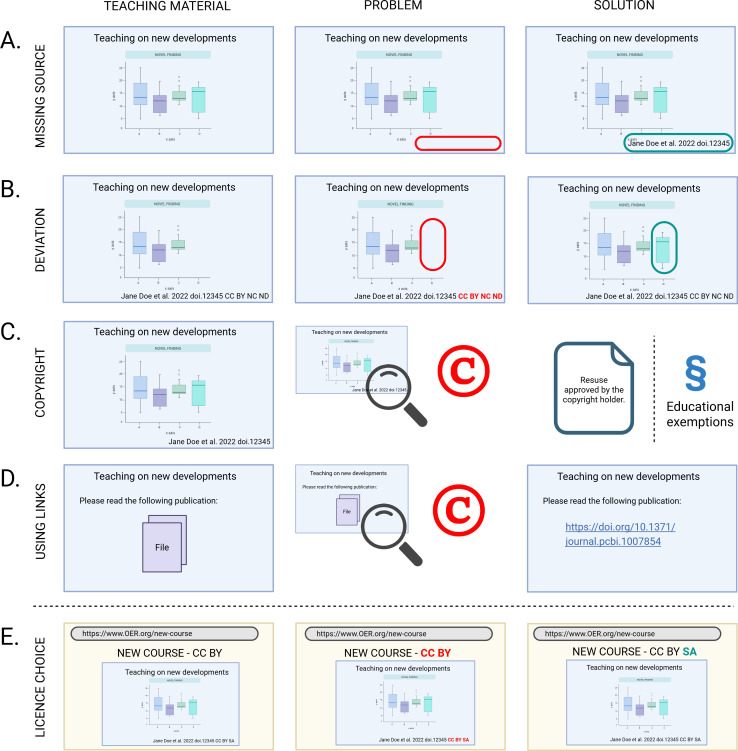
Examples of legal challenges and solutions in FAIR OER. The figure provides an overview of different legal pitfalls that educators may encounter when creating and sharing OER, and how to avoid them. The content of the examples is addressed throughout the paper. Created in Biorender.com.

To fully realize the potential of OER, it is essential that materials are findable, accessible, interoperable, and reusable (FAIR) [2]. FAIR data principles facilitate dissemination and reuse, thereby accelerating advancement, fostering collaboration, and maximizing the impact of published work—both for scientific findings and OER [3,4]. However, there are challenges when creating FAIR OER with reused materials while respecting legal rights, namely copyright and legally secure reuse of materials for educational purposes [5,6] (Fig 1).

**Fig 2:**
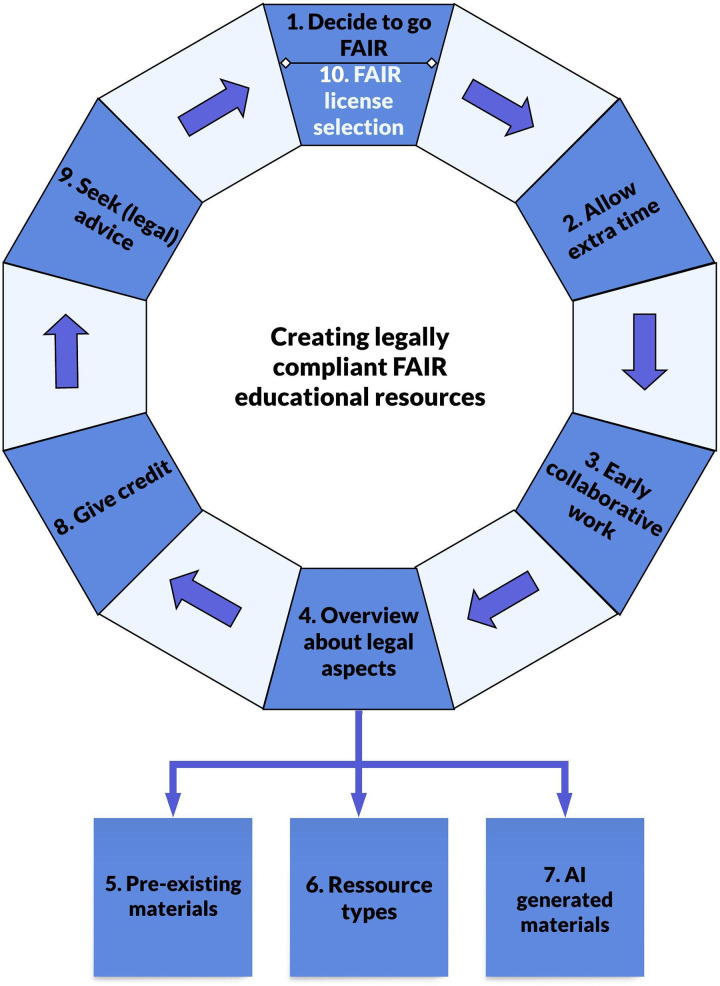
Ten quick tips for creating legally compliant FAIR educational resources. The quick tips outline key steps from conceptualization to publication, highlighting legal considerations, collaboration, and resource management. Created in Lucid (lucid.co).

In this article, we aim to share learning points from the process of producing and sharing FAIR online OER. Given the diversity of legal frameworks across different countries, it should be noted that this article cannot be considered legal advice but rather outlines strategies and pitfalls to avoid in the context of legally sound FAIR OER. Overall, our goal is to inspire more scholars to create FAIR educational resources without the fear of breaking the law. At the same time, our objective is to make academics aware of the copyright issues involved in open education. The following 10 quick tips will guide the process of creating legally compliant FAIR open educational materials, covering all stages from the initial decision to adopt FAIR practices to the final steps of properly attributing and licensing educational content (Fig 2). The quick tips identify key decision points to consider in the planning and execution of publishing FAIR OER to ensure legal certainty. While Garcia and colleagues provide a comprehensive approach to making training materials FAIR in their entirety [4], this publication specifically focuses on legal aspects.

### 1. Actively decide to go FAIR

Successful FAIR implementation can be challenging and therefore demands a conscious and coordinated decision of the content creation. Educators should work closely with the appropriate stakeholders, such as institutional leadership and administrative departments to ensure full support, resource allocation, and organizational buy-in. During the decision-making process, it is recommended to reflect both the benefits of OER and how potential challenges will be addressed. This article discusses several of these challenges and how they can be addressed. Benefits include the improved resource efficiency by minimizing redundant work, and promotion of inclusivity both in terms of accessibility and content relevance, ensuring high-quality educational materials accessible regardless of socioeconomic status [7,8]. Potential co-creators should also be involved early on to ensure a shared commitment to FAIR principles and sustain collective effort (tip 3). A strong commitment to go FAIR in the beginning and planning accordingly will ultimately save time and resources throughout the project.

### 2. Allow extra time and resources for making your FAIR educational resources legally secure

Navigating legal requirements and understanding licensing information takes energy and time. Educators might need to adapt previous workflows and seek additional support to ensure the legal compliance of FAIR educational resources.

It is advisable to declare your intention to develop FAIR OER when applying for funding. This approach allows for the inclusion of additional budget for critical elements that support legal soundness, such as budgeting for legal advice (tip 9). It may also be sensible to allocate funds and time for initial training to minimize potential misunderstandings during project implementation (tip 3) and reduce the risk of infringing non-open materials.

Also, involve university libraries as they have extensive experience in reviewing materials for adequacy and legal status. Especially in cases of limited funding, obtaining support from institutional resources and proactive planning at the beginning of the project are key.

Despite the challenges posed by legal complexities, investing in legal compliance early on is a worthwhile investment. Once a workflow is established and legal knowledge is acquired, these processes can be streamlined, making future projects more efficient.

### 3. Get your collaborators involved at an early time point

Educational projects often are of collaborative nature. This necessitates early engagement with all parties involved to ensure buy-in and to address legal challenges effectively. Collaborators should be informed of potential legal challenges, strategies for compliance, and additional workload early in the project. This proactive approach minimizes the risk of encountering legal hurdles later and ensures a smooth workflow throughout the project. Otherwise, there is a risk that the OER created will not be legally compliant due to a lack of awareness of the contributors, requiring additional cataloguing and usability assessment (tip 6).

Special caution should be given to cross-country collaborations as national legislations apply and standardized international copyright does not exist [9]. For example, copyright in the European Union focuses on the rights of the creator of a material, whereas copyright as practiced in the US and the Commonwealth emphasizes the financial interest of the publisher, representing two very different approaches [10]. In this case, it is important to communicate differences and to find a common determinator before starting to create materials, especially if the materials are aimed to be published by institutions in multiple countries.

### 4. Get an overview about legal aspects to consider

Grasping the basics of copyright law in one’s country is essential, as local laws will apply in legal disputes. Copyright laws protect intellectual property but also impose restrictions on knowledge dissemination [9]. Most national copyright laws allow exceptions for educational purposes (Fig 1C) [11]. However, these are typically limited to classroom use or restricted access, making them unsuitable for FAIR OER [12]. Therefore, it is essential to familiarize oneself with licensing options.

**Fig 3:**
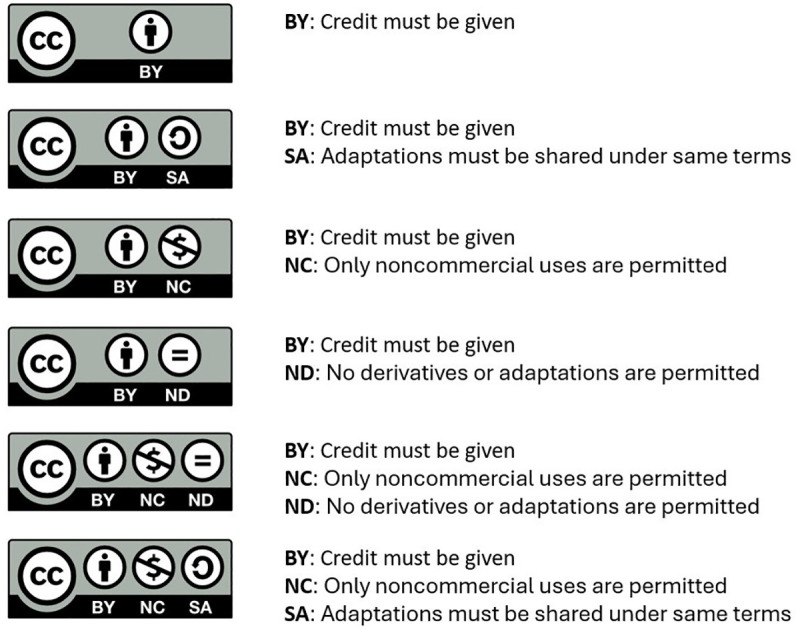
Overview of available Creative Common Licenses and their conditions. The Silence icons are by Font Awesome, used under CC BY 4.0. Figure inspired by [13].

**Fig 4:**
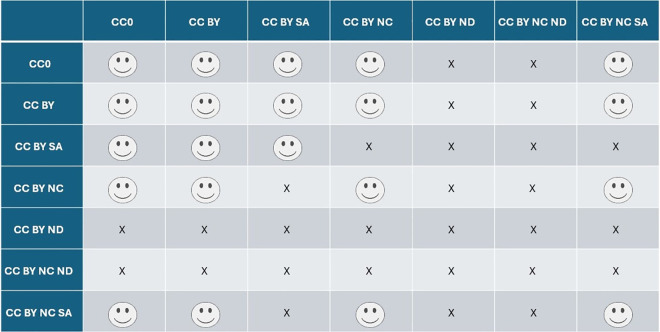
Overview of Creative Common License compatibilities. This figure shows the compatibility of different Creative Commons (CC) licenses for use in educational resources. Whether or not two different licenses are compatible can be determined by choosing one license (e.g., CC BY SA) in the rows and the other license (e.g., CC BY NC) in the columns. A smiley icon (☺) indicates a compatible license, while an “X” indicates, that the two licenses cannot be used together. Inspired by CC License Compatibility Chart [17].

The most commonly used and globally recognized Creative Common Licenses, offer five main licenses, each with specific terms for use (Fig 3) [13]. In addition, there is the Public Domain Dedication (CC0), which functions as the Creative Commons equivalent of public domain rules, where the copyright holder explicitly relinquishes copyright, as this is not possible in all legislations [14,15]. Whereas Creative Commons licenses and other licensing schemes facilitate the reuse of materials, they do not imply unrestricted use. Each license comes with distinct requirements that must be met to avoid copyright infringement (Fig 1B) [13,16]. Additionally, understanding license compatibility and version differences is crucial as these conditions might further limit the reuse and mixing of materials (Fig 4). Adhering to these specific conditions is not only a legal requirement, but respects the original creator’s intent, ensuring that their work is used fairly and in accordance with the permissions they have granted.

### 5. Address pre-existing materials in your project

Oftentimes, OER emerge from pre-existing materials, minimizing the resource investment required for creating FAIR OER. However, this approach also involves specific challenges.

**Fig 5:**
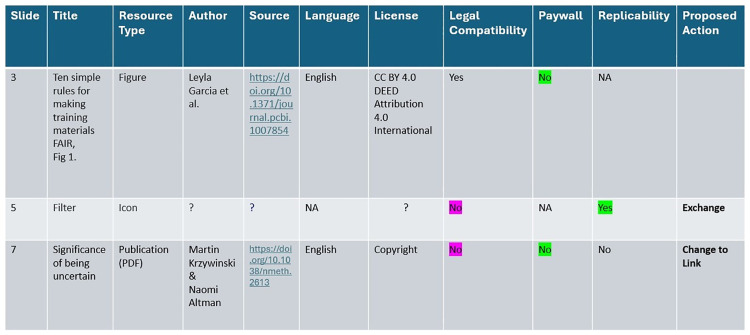
Cataloguing of course materials. This figure catalogues (pre-existing) course materials, helping transform them into FAIR and legally compliant resources. Parameters included, like License or Paywall help to facilitate management and decision-making as per tips 5 and 6. Further information and examples can be found in the Supporting information. To enhance readability, color coding can be employed.

**Fig 6:**
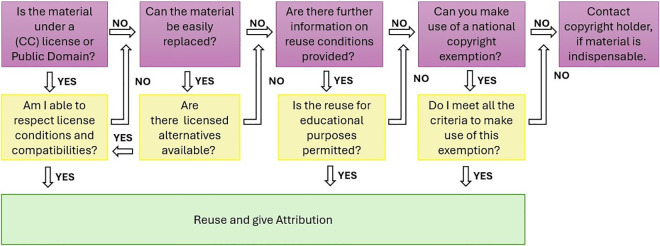
Workflow for Assessing (pre-existing) Course Materials for FAIR Transformation. This decision tree outlines a workflow for evaluating course materials for FAIR transformation.

To ensure legal compliance, it is essential to catalog and track all resources utilized in previously created materials (Fig 5). This includes verifying the licensing and copyright status of each material used. Understanding the legal constraints on these resources might not always be straightforward. Ideally, the source of a reused material is known. If it is not immediately apparent, a simple online search may help. For images, the function reverse image search, which is included in many search engines, can be helpful. Journal articles often include information about copyright or licensing status either at the top or bottom of the publication. Websites typically provide a copyright statement at the bottom or in the imprint section. Videos often provide information at the end of the description. If no specific statement is found, it has to be assumed that copyright applies, as the absence of a copyright notice does not negate the existence of copyright protection [18]. The same applies if a material cannot be tracked back to its original source. In such cases, it is safer to either avoid using the material or seek a suitable replacement (Fig 6).

### 6. Navigate diverse resource types

From images to scientific figures, each resource type presents unique challenges and considerations in terms of copyright compliance.

Small or seemingly interchangeable resources, such as icons or images, generally carry copyright protection unless indicated otherwise, but can usually be easily replaced by licensed alternatives. More challenging are non-exchangeable resources, such as a graph from a copyrighted scientific publication. It may not be legally possible to reuse this graph for OER, even though it is essential. However, it is important to understand that copyright protects the copying of materials—not the reference. Therefore, one strategy to avoid copyright infringement is to include a hyperlink or embed a video (Fig 1D) [19]. This allows students to access important information without duplication. One pitfall, however, are paywalls as they might hinder access. The browser extension ‘unpaywall’ can offer a solution by finding free, legal versions of paywalled research articles [20].

### 7. Keep up to date with AI developments

An emerging type of resource is artificial intelligence (AI) generated material unlike traditional works, AI-generated materials may not be eligible for copyright protection, raising important questions about ownership and use [21]. To ensure legal compliance and uphold ethical standards in FAIR OER, it is crucial to review the terms of use of any AI tool used to generate content, as some platforms may retain rights to the content or impose restrictions. Relevant information can mostly be found in the copyright or content section of “terms & use”. Even when AI-generated content is not protected by copyright, proper attribution to the AI tool or platform should be provided, and any specified conditions of use must be respected [15]. However, a recent review of various terms and conditions of use indicates that most AI tools have liberal rules regarding the re-use of generated output. OpenAI, the provider of ChatGPT, for example, only prohibits the use of output to harm others and all output is owned by the user of the tool [22]. However, educators should be cautious with AI-generated content, as its origins may include copyrighted material, potentially leading to copyright infringement [23]. Additionally, users should be aware that any uploaded content may be stored and shared by AI tools, which could have legal consequences for the user responsible for the initial upload, if it includes copyrighted material, as they remain its owner [22].

Given the complexities surrounding AI-generated content—such as unclear copyright status, platform-specific terms, often unknown user responsibilities, and the novel nature of this resource—it is important to continuously review current developments in this dynamic field. Further, documentation of the findings might be considered.

### 8. (Always) give credit

Proper attribution is a fundamental aspect of copyright compliance in educational projects and is required for quotation and most licenses. Therefore, the original source and author should always be provided (Fig 1A). However, attribution requirements vary based on licensing conditions (tip 4), with, e.g., Creative Commons licenses typically mandating attribution including mentioning of the title, author, original source and the exact CC license, preferably with a link to the terms of the specific license [24,25]. Incorporating attribution information prominently alongside reused materials or, if not possible in metadata, ensures compliance with licensing conditions as non-adherence might lead to invalidation of in general reusable licenses (Table 1).

**Table 1:** Creative Common 4.0 metadata attribution template.

^Slide^	^Title^	^Author^	^Source^	^License^	^Link^	^Comment^
3	Ten simple rules for making training materials FAIR, Fig 1. Ten simple rules for making training materials FAIR.	Leyla Garcia and colleagues	https://doi.org/10.1371/journal.pcbi.1007854	ATTRIBUTION 4.0 INTERNATIONAL CC BY 4.0 DEED	CC BY 4.0 Deed | Attribution 4.0 International | Creative Commons	
5	A manifesto for reproducible science Fig 1. Threats to reproducible science.	Marcus R. Munafò and colleagues	https://doi.org/10.1038/s41562-016-0021	ATTRIBUTION 4.0 INTERNATIONAL CC BY 4.0 DEED	Deed - Attribution 4.0 International - Creative Commons	
9	Finding the best fit for improving reproducibility: reflections from the QUEST Center for Responsible Research.	Drude and colleagues	https://doi.org/10.1186/s13104-022-06108-x	ATTRIBUTION 4.0 INTERNATIONAL CC BY 4.0 DEED	Deed - Attribution 4.0 International - Creative Commons	

The suggested structure of the template includes all the attribution information required for license compliance and can be added to the metadata of a course.

### 9. When in doubt—seek (legal) advice and protect yourself

Although educational projects might seem unlikely targets for legal disputes, taking proactive measures can help prevent potential conflicts. Consulting legal experts or knowledgeable colleagues, such as library staff can provide clarity and guidance in navigating complex legal issues. For large-scale OER projects, professional advice is particularly valuable. When choosing legal counsel, it is important to find a lawyer with a suitable focus and expertise in country-specific copyright but also licensing, and if needed international copyright laws. Since lawyers with this niche expertise are relatively rare, seeking recommendations from colleagues and project partners, or searching professional networks like LinkedIn, may be helpful.

This proactive approach can prevent undesirable outcomes, such as having to withdraw OER after significant investment, thereby preserving the intended impact and visibility of high-quality resources or having to pay a fine to the copyright holder.

### 10. Decide under which license your work should be offered

License selection determines the terms under which materials can be reused and redistributed, influencing their impact and accessibility as different licenses offer varying degrees of flexibility. Selecting a license that balances openness with legal compliance involves assessing project objectives and licenses of re-used materials. Certain re-used materials within the created OER might prevent the choosing of the desired license (e.g., CC BY SA) as all license conditions must be respected and compatibility ensured (Fig 1E). If possible, for maximal re-use and re-distribution, the most liberal CC license CC-BY is advisable. It is also possible to apply different licenses to individual slides or modules within the OER, which allows greater flexibility when combining original and third-party content with varying license requirements (Fig 4). Providing clear licensing information ensures transparency and facilitates lawful reuse of materials, increasing the possibility of credited distribution of works.

## Don’t give up—FAIR is worth it

Legal rights can indeed be overwhelming. However, the ability to create OER that adhere to FAIR principles and copyright laws is a potent tool for disseminating knowledge and reaching diverse audiences. By ensuring compliance with legal requirements, educators can promote open science ideals and expand access to educational resources. This approach is particularly significant for resource-limited countries, where adhering to FAIR principles can bridge educational gaps and provide equitable access to quality learning materials.

Following the tips mentioned above is a first important step toward understanding and integrating legal considerations into educational projects. As you continue to apply these guidelines, you’ll quickly establish a routine that saves time and increases efficiency in all future projects.

To further support your journey, consider exploring additional resources that provide guidance on legal compliance and the creation of FAIR materials. Some valuable starting points include: creativecommons.org, UNESCO Open Educational Resources (unesco.org/open-educational-resources), as well as the Tables and Figures provided within this paper, which offer a breakdown of key legal considerations.

Empower yourself with the legal knowledge necessary to create FAIR educational resources. It may require effort and dedication, but the rewards of reaching broader audiences and advancing open science are well worth it.

## Supporting information

S1 Text Supporting Material. This file provides example presentation slides outlining legal difficulties and illustrating how the proposed Table in Fig 5 for assessing pre-existing materials can be used.
(PDF)
