# Supplementary material for: Ten quick tips for navigating intellectual property in FAIR educational resources
Source: PLoS Comput Biol. 2025 Jul 8;21(7):e1013208. doi: 10.1371/journal.pcbi.1013208 (PMC12237045; doi:10.1371/journal.pcbi.1013208)
Supplement: S1 Text — This file provides example presentation slides outlining legal difficulties and illustrating how the proposed Table in Fig 5 for assessing pre-existing materials can be used. (PDF) [file pcbi.1013208.s001.pdf]

Supporting Information

Here we illustrate the assessment and transformation of pre-existing course materials into FAIR OER with examples using our suggested table. Examples of three presentation slides are shown. These examples correspond to Figure 5. Please note that the example slides are not intended to represent pedagogically valuable or content-rich teaching materials. They merely serve to schematically illustrate types of resources and legal considerations.

Slide 3

Steps to take in order to create FAIR teaching materials

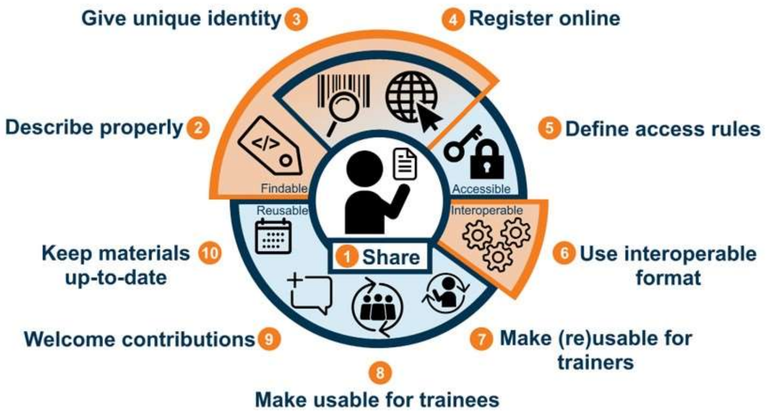

Ten simple rules for making training materials FAIR, Fig 1. Ten simple rules for making training materials FAIR., Leyla Garcia et al. ,CC BY 4.0, https://creativecommons.org/licenses/by/4.0/

This slide shows a best practice example of legal compliance and attribution. The attribution with all the necessary information is included at the bottom of the slide. The CC BY 4.0 license can be used and combined without restriction as long as attribution is provided, as in this example.

Slide 5

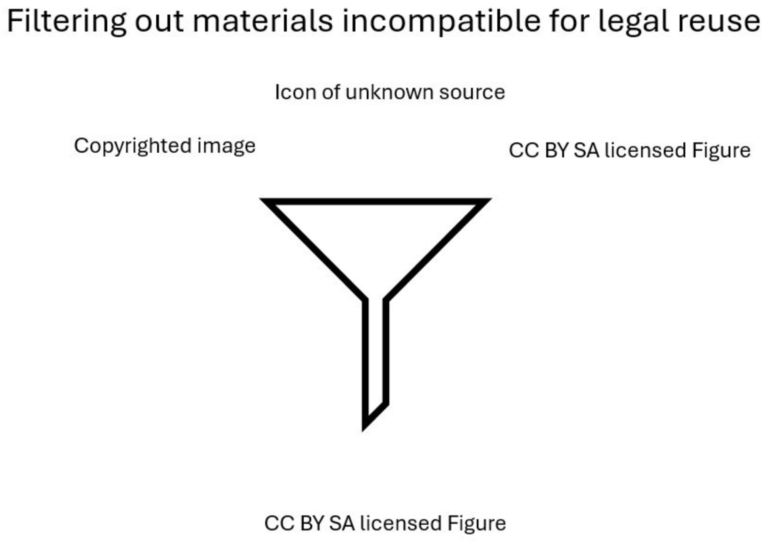

This slide illustrates a common problem with existing materials, namely the lack of source information. Often educational materials are created for classroom use, perhaps with less attention to source attribution, especially for replaceable elements such as images or icons. However, when such material is transformed into OER, its legal soundness needs to be ensured.

Looking at the type of resource in the proposed table can help to identify which resources may be easily interchangeable, and what strategies may be available for modification. The replicability of a resource can also be indicated in a separate column of the table.

(The filter icon used in this slide was made available under Public Domain (CC0).)

Slide 7

To learn more, please read the following article

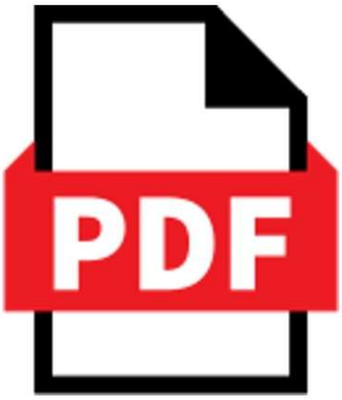

This slide provides students with a PDF for further reading. However, this publication is protected by copyright (see Figure 5 column “License”). Therefore, creating and sharing a PDF is not allowed. A good way to make the information available is to provide a link. It is important to know whether a paywall might prevent students who are not affiliated to a university with certain privileges, from accessing the publication. If a paywall exists, this can also be documented in our suggested table.

To summarize the use of the proposed table: The most important information in the table is certainly legal compliance (Legal Compatibility). If there are resources in the current version of the educational material that are not legally compatible with an OER, action must be taken to ensure legal compliance in the FAIR OER. The questions can be asked whether it is replaceable (Replicability) and whether there is a paywall, in case a link can be provided (Paywall). Depending on the answers to these questions, strategies can be developed to deal with the legal incompatibility (Proposed Action).

Additional information such as language can be helpful if a national course is to be transformed into an international material. Information such as author, source, title, slide can already prepare the creation of a metadata table as shown in Table 1.

Below we present solutions to the problems identified in slides 5 and 7.

Slide 5

Filtering out materials incompatible for legal reuse

Icon of unknown source

Copyrighted image

CC BY SA licensed Figure

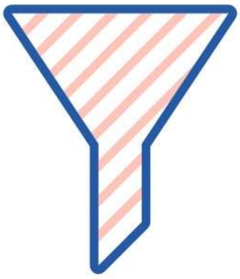

CC BY SA licensed Figure

Toicon-icon-hatch-filter.svg, Carol Liao/toicon.com, https://commons.wikimedia.org/wiki/File:Toicon-icon-hatch-filter.svg, CC BY 4.0,https://creativecommons.org/licenses/by/4.0/deed.en

The unknown icon has been replaced by a known source with a compatible license and attribution according to the license terms.

Slide 7

To learn more, please read the following article

https://www.nature.com/articles/nmeth.2613

The PDF can be replaced by a link, since there is no paywall and the article in question is freely accessible.
